# CHK1 is an integral regulator of DNA replication in human cells

**DOI:** 10.1038/s41419-026-08624-1

**Published:** 2026-03-26

**Authors:** Siting Li, Dandan Zhu, Mengfan Tang, Min Huang, Xu Feng, Litong Nie, Huimin Zhang, Ling Yin, Sarah Keast, Chang Yang, Tiantian Ma, Junjie Chen

**Affiliations:** 1 Department of Experimental Radiation Oncology, The University of Texas MD Anderson Cancer Center, Houston, TX USA; 2 Department of Immunology, School of Medicine, Nanjing University of Chinese Medicine, Nanjing, China

**Keywords:** DNA replication, Cancer therapy

## Abstract

CHK1, a key serine/threonine kinase, is essential for cell cycle progression and genome maintenance in response to DNA damage and/or replication stress. However, its functions during normal DNA replication remain to be defined. Here, we employed the dTAG system to achieve rapid and selective CHK1 depletion in cells and examined the consequences of its acute loss. CHK1 degradation led to rapid cell death, with significant loss of viability within 16 h and complete lethality by 48 h, indicating critical roles of CHK1 during normal DNA replication. Rescue experiments demonstrated that only full-length, catalytically active CHK1 could restore cell survival, emphasizing the essential role of its kinase function and ATR-dependent phosphorylation. CHK1 depletion triggered extensive DNA damage, as evidenced by increased γH2AX and RPA2 phosphorylation, and caused S-phase arrest, replication fork collapse, and failure to enter mitosis. Interestingly, cells arrested at the G1/S boundary, which do not undergo DNA replication, were still sensitive to CHK1 depletion. These data reveal a critical role of CHK1 in suppressing replication fork progression even in the absence of DNA replication. Thus, our results highlight CHK1’s indispensable role in the management of replication fork stability and cell cycle progression, providing a refined mechanistic understanding of its function during normal cell proliferation.

## Introduction

Genomic stability is fundamental to cellular and organismal survival and to the prevention of diseases such as cancer. To maintain genome integrity, cells have evolved sophisticated surveillance and repair mechanisms that detect DNA damage and ensure accurate replication and faithful transmission of genetic material [1]. A central component of this network is the ATR-CHK1 signaling pathway, which plays a critical role in safeguarding replication fork stability and preventing the accumulation of deleterious mutations during DNA replication [2].

CHK1, a serine/threonine kinase, functions as a key downstream effector of ATR, coordinating cellular responses to replication stress and DNA damage. Through phosphorylation of multiple substrates involved in replication fork stabilization, checkpoint activation, and DNA repair, CHK1 integrates signaling pathways that preserve genome integrity [2]. Disruption of the Treslin–CHK1 interaction leads to increased initiation of chromosomal DNA replication during an unperturbed cell cycle, establishing CHK1 as a negative regulator of origin firing [3]. Under conditions of replication stress, CHK1 promotes replication fork stabilization and delays mitotic entry, thereby preventing catastrophic genomic instability [4]. The importance of CHK1 is further underscored by its relevance to cancer therapy, as tumor cells experiencing elevated endogenous replication stress display heightened sensitivity to CHK1 inhibition [5]. Consistently, inhibition of CHK1 results in the accumulation of single-stranded DNA and induction of DNA strand breaks [6]. Genetic depletion or pharmacological inhibition of CHK1, therefore, leads to DNA damage accumulation and synthetic lethality in cancer cells [7].

Although small-molecule CHK1 inhibitors, such as UCN-01 and AZD7762, have provided important insights into CHK1 function, their interpretation is often confounded by off-target effects and incomplete or indirect inhibition of CHK1 activity [8]. Consequently, more precise experimental approaches are required to define the essential roles of CHK1, particularly during normal cell-cycle progression. To directly address how CHK1 functions during unperturbed DNA replication, we employed the dTAG system, a highly selective and temporally controlled protein degradation strategy, to achieve acute depletion of CHK1 in human cells. This approach enables rapid elimination of CHK1 at defined cell-cycle stages, thereby allowing precise examination of the immediate cellular consequences of CHK1 loss.

Using this system, we sought to dissect the mechanistic roles of CHK1 during normal DNA replication, cell-cycle progression, and cell viability. Specifically, we aimed to determine whether CHK1 function in cell-cycle progression is strictly dependent on its kinase activity and ATR-mediated phosphorylation. By leveraging the temporal control afforded by the dTAG system, our study provides new insight into the essential roles of CHK1 in maintaining DNA replication fidelity and genome stability.

## Results

### CHK1 depletion leads to cell lethality

CHK1 is a critical regulator of the replication stress response, ensuring proper DNA synthesis and cell-cycle progression in response to DNA damage and replication stress, particularly during S phase. Previous studies have established that CHK1 inhibition or depletion results in replication fork stalling, accumulation of DNA damage, and eventual cell death [9–11].

However, the precise kinetics of these events and their dependence on CHK1 activity remain incompletely understood. Several key questions, therefore, remain unresolved. First, is CHK1 required during every S phase and/or cell cycle? In other words, does CHK1 depletion lead to immediate loss of viability, or does it cause a progressive accumulation of DNA damage and replication stress over multiple S phases and/or cell cycles that ultimately culminates in cell lethality? Second, is basal CHK1 activity sufficient to sustain cell viability, or is stress-induced activation of CHK1 essential? Third, what are the critical functions of CHK1 at replication forks under normal versus stressed conditions?

To address these questions, we employed the dTAG system to achieve acute and selective degradation of CHK1 in human 293A cells (dTAG-CHK1) and systematically analyzed the cellular consequences at multiple time points following CHK1 loss.

**Fig. 1:**
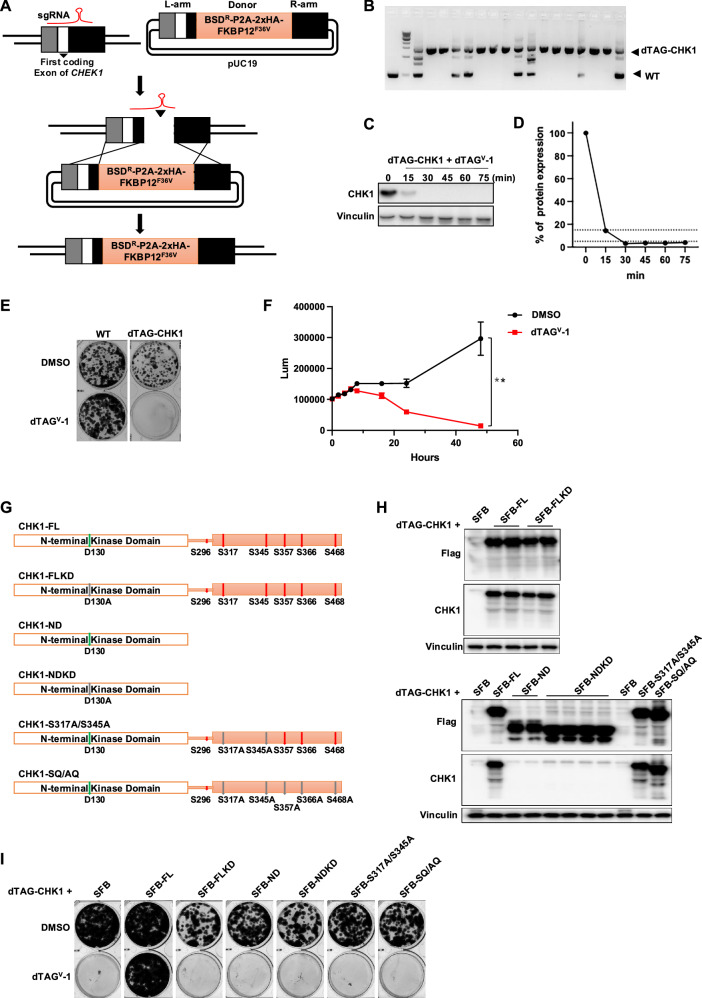
Rapid depletion of using the degradation tag (dTAG) system. **A** Schematic illustration of the strategy used to generate the dTAG-CHK1 knock-in cell line. FKBP12^F36V^ and an HA tag were inserted in frame at the endogenous *CHEK1* locus using CRISPR/Cas9-mediated homologous recombination. **B** Genotyping PCR analysis confirming successful insertion of the dTAG cassette at the *CHEK1* locus. **C** Immunoblot analysis showing time-dependent degradation of CHK1 protein following treatment with dTAG^V^-1 (1 μM). **D** Quantification of CHK1 protein levels from panel C, normalized to vinculin and plotted as the percentage of remaining CHK1 expression over time. **E** Representative images of colony formation assays. Wild-type (WT) and dTAG-CHK1 cells were treated with DMSO or dTAG^V^-1 for 6 days. **F** Cell viability of dTAG-CHK1 cells measured by a luminescence-based ATP assay over a 48-h time course in the presence or absence of dTAG^V^-1 (*n* = 3). *p* < 0.05. **G** Schematic representation of CHK1 constructs used for rescue experiments, including full-length CHK1 (FL), full-length kinase-dead CHK1 (FLKD), N-terminal kinase domain (ND), kinase-dead N-terminal domain (NDKD), and ATR phosphorylation-deficient mutants (S317A/S345A and SQ/AQ). **H** Immunoblot analysis confirming expression of endogenous dTAG-CHK1 and exogenous SFB-tagged CHK1 WT and mutant constructs. **I** Colony formation assays in dTAG-CHK1 cells expressing the indicated CHK1 constructs and treated with DMSO or dTAG^V^-1.

The dTAG system pairs a small-molecule degrader specific for FKBP12^F36V^ with expression of FKBP12^F36V^ fused in-frame to a protein of interest [12]. CRISPR/Cas9-mediated gene editing enables the insertion of defined DNA fragments (e.g., epitope tags and drug-resistance markers) at endogenous gene loci through sequence-specific double-strand break induction followed by homology-directed repair [13–15]. Using this knock-in strategy, we generated human 293A-derived cells expressing a dTAG cassette (BSD^R^-P2A-2xHA-FKBP12^F36V^) at the N terminus of the endogenous CHK1 locus, thereby establishing a conditional CHK1 knockout cell line (hereafter referred to as dTAG-CHK1 cells) (Fig. 1A, B). We then used dTAG^V^-1, a highly selective VHL-recruiting dTAG molecule [16], to induce rapid degradation of FKBP12^F36V^-tagged CHK1. As shown in Fig. 1C, D immunoblot analysis revealed that treatment with 1 µM dTAG^V^-1 resulted in >75% CHK1 depletion as early as 15 min, with near-complete degradation observed within 30 min. Accordingly, all subsequent experiments were performed using 1 µM dTAG^V^-1 to induce acute CHK1 degradation in dTAG-CHK1 cells.

Here, we performed cell proliferation assays to determine whether acute CHK1 depletion by dTAG^V^-1 in dTAG-CHK1 cells leads to cell lethality. Parental 293A wild-type (WT) cells were used as controls, and neither dTAG^V^-1 nor DMSO treatment affected cell viability in WT cells. In contrast, virtually no dTAG-CHK1 cells survived following 10 days of dTAG^V^-1 treatment, whereas DMSO-treated dTAG-CHK1 cells remained viable (Fig. 1E).

To further assess the kinetics of cell death following CHK1 degradation, we performed CellTiter-Glo viability assays (Fig. 1F). A significant reduction in cell viability was observed as early as 16 h after dTAG^V^-1 treatment. By 24 h, approximately two-thirds of the cells had lost viability, and by 48 h, near-complete cell death was observed. These results demonstrate that acute CHK1 depletion using the dTAG system leads to rapid cell lethality, suggesting that loss of CHK1 function may be sufficient to trigger cell death within a single cell cycle. Consistent with previous reports showing that CHK1 loss is lethal in proliferating cells [17, 18], our study provides enhanced temporal resolution, revealing that substantial loss of viability occurs within 16 h and progresses to near-complete lethality by 48 h (Fig. 1F).

To further confirm that the observed lethality in CHK1-depleted cells was specifically attributable to loss of endogenous CHK1, we re-expressed full-length CHK1 in dTAG-CHK1 cells via lentiviral transduction. CHK1 is a serine/threonine kinase composed of an N-terminal kinase domain that mediates catalytic activity and a C-terminal regulatory region containing multiple phosphorylation sites required for CHK1 activation in response to DNA damage and replication stress [19]. The D130 residue, located within the ATP-binding pocket of the kinase domain, is essential for CHK1 catalytic activity, and mutation of this residue generates a kinase-dead form of the protein [20, 21].

The C-terminal regulatory domain contains five conserved SQ motifs (ATR-targeted glutamine-adjacent serine residues), including S317, S345, S357, S366, and S468, which are phosphorylated in response to replication stress and DNA damage and are critical for CHK1 activation and checkpoint signaling [22]. In addition, S296 is an autophosphorylation site that contributes to CHK1 stability and function, with phosphorylation at this site promoting CHK1 degradation through the SCF-βTrCP ubiquitin ligase pathway [23]. CHK1 is thought to be maintained in an autoinhibited conformation through intramolecular folding of its C-terminal domain, thereby restricting kinase activity until ATR-dependent phosphorylation relieves this inhibition [24]. Based on these regulatory features, we generated a series of CHK1 truncation and point mutants to define the domains and phosphorylation events required for CHK1 function in maintaining cell viability.

As shown in Fig. 1G, we generated the following CHK1 constructs: (1) CHK1-FL, the full-length WT CHK1; (2) CHK1-FL(KD), the full-length catalytically inactive CHK1 carrying the D130A point mutation; (3) CHK1-ND, an N-terminal kinase domain fragment (amino acids 1-265); (4) CHK1-ND(KD), an N-terminal kinase domain fragment harboring the D130A mutation; (5) CHK1-S317A/S345A, a full-length CHK1 mutant with alanine substitutions at two key ATR phosphorylation sites; and (6) CHK1-SQ/AQ, a full-length CHK1 mutant with alanine substitutions at five ATR phosphorylation sites (S317A, S345A, S357A, S366A, and S468A). Together, these constructs allowed us to define which CHK1 domains and regulatory modifications are required to support cell survival in the absence of endogenous CHK1.

Expression of endogenous dTAG-CHK1 and exogenous SFB-tagged CHK1 variants (full-length, truncated, or point mutants) were assessed by immunoblotting using antibodies against CHK1 and FLAG (Fig. 1H). All exogenous CHK1 proteins were detected with the FLAG antibody, whereas only full-length CHK1 proteins (WT or mutants) were recognized by the CHK1 antibody, which detects epitopes within the C-terminal region of CHK1. dTAG-CHK1 cells were treated with dTAG^V^-1 to induce degradation of endogenous CHK1, with DMSO-treated cells serving as controls.

As shown in Fig. 1I, CHK1 depletion resulted in pronounced loss of cell viability, whereas re-expression of full-length WT CHK1 efficiently rescued cell survival. In contrast, truncated CHK1 fragments (kinase domain alone), catalytically inactive full-length CHK1 (D130A), or phosphorylation-deficient CHK1 mutants (S317A/S345A or SQ/AQ) failed to restore viability. These results demonstrate that CHK1-mediated cell survival requires its full-length structure, intact kinase activity, and ATR-dependent phosphorylation, extending previous studies emphasizing the kinase-dependent functions of CHK1 [25].

Notably, the inability of the N-terminal kinase domain alone to support cell proliferation suggests that the C-terminal region of CHK1 contributes functions beyond simple autoinhibition, challenging the prevailing model that the C terminus acts solely as a negative regulatory domain [24]. Moreover, the failure of phosphorylation-deficient CHK1 mutants to rescue viability indicates that basal ATR-dependent phosphorylation of CHK1 is essential for cell survival. Collectively, these findings support two conclusions: (i) both basal and stress-responsive CHK1 activities are required for cell proliferation, and (ii) the C-terminal domain of CHK1 plays previously underappreciated roles in sustaining cell viability.

### CHK1 depletion leads to replication stress, DNA damage accumulation, and impaired cell-cycle progression

**Fig. 2:**
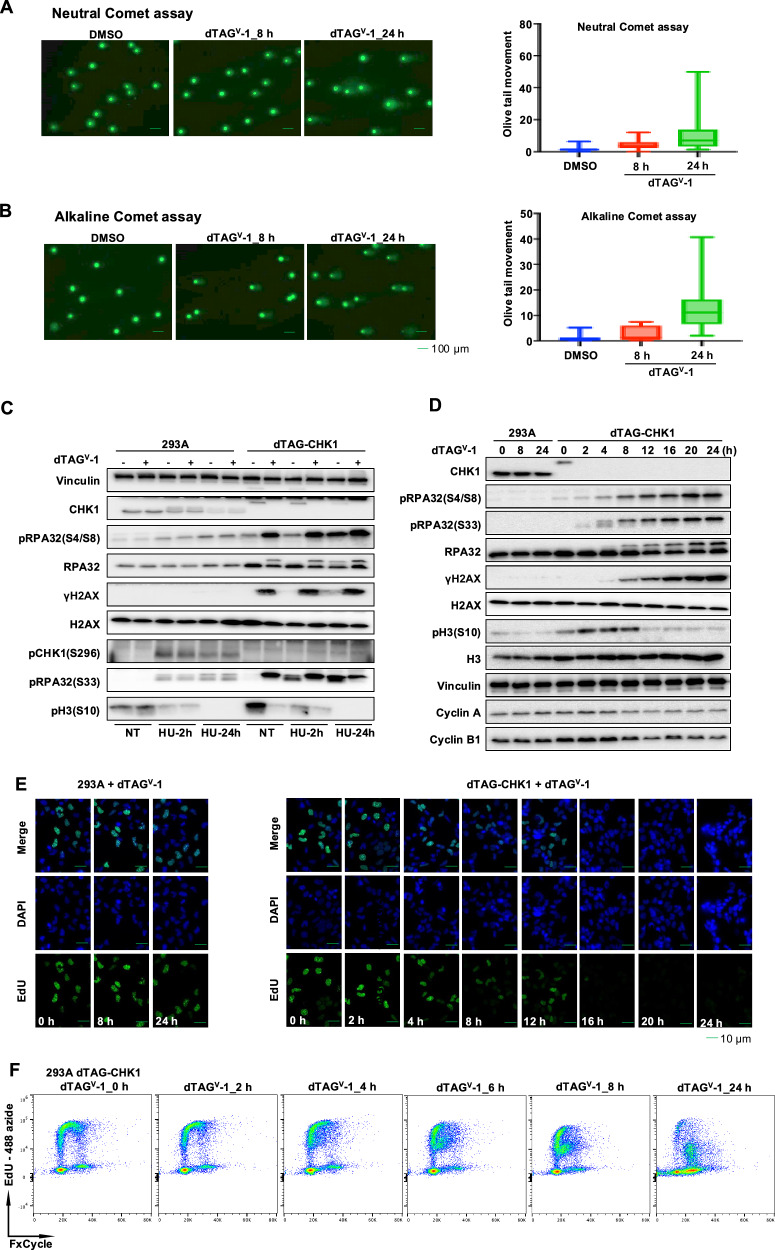
CHK1 depletion induces DNA damage and disrupts DNA replication. **A**, **B** Neutral (**A**) and alkaline (**B**) comet assays showing DNA damage in dTAG-CHK1 cells treated with dTAG^V^-1 for 8 or 24 h. Quantification of comet tail moments is shown in the right panels. **C** Immunoblot analysis of DNA damage and replication stress markers in parental 293A and 293A-derived dTAG-CHK1 cells treated with dTAG^V^-1 or hydroxyurea (HU) for 2 or 24 h. **D** Time-course immunoblot analysis of dTAG-CHK1 cells treated with dTAG^V^-1 for the indicated durations, showing progressive accumulation of DNA damage markers and a reduction in pH3(S10) and cell-cycle-associated proteins. **E** EdU incorporation assays in parental 293A and 293A-derived dTAG-CHK1 cells treated with dTAG^V^-1. DNA synthesis was monitored at the indicated time points by EdU labeling and DAPI staining. **F** Flow cytometry analysis showing EdU incorporation and DNA content (FxCycle) in dTAG-CHK1 cells following dTAG^V^-1 treatment for the indicated durations.

To assess the impact of CHK1 depletion on genomic integrity, we performed both neutral and alkaline comet assays in dTAG-CHK1 cells treated with 1 µM dTAG^V^-1. As shown in Fig. 2A, neutral comet assays revealed a time-dependent increase in DNA double-strand breaks, as evidenced by elongated comet tails at 8 h and a further increase at 24 h following treatment. Consistently, alkaline comet assays, which detect both single- and double-stranded DNA breaks, demonstrated even more pronounced DNA damage at the corresponding time points, indicating that acute CHK1 depletion induces extensive genomic instability (Fig. 2B).

To further examine the relationship between CHK1 loss and replication stress–associated DNA damage, we compared parental 293A and dTAG-CHK1 293A cells under conditions of hydroxyurea (HU) treatment, which induces replication fork stalling. Cells were treated with 2 mM HU for 2 or 24 h in the presence of dTAG^V^-1 (+) or DMSO (−), and the levels of key DNA damage and replication stress markers were assessed by immunoblotting (Fig. 2C).

In parental 293A control cells, HU treatment induced only weak to modest increases in γH2AX and phosphorylation of RPA2 at both S4/S8 and S33 sites, consistent with a mild replication stress response. CHK1 protein levels were unaffected in these cells following HU or dTAG^V-^1 treatment, and phosphorylation of CHK1 at S296 was readily detected, indicating intact ATR-dependent activation of CHK1 in response to HU. In contrast, dTAG-CHK1 cells exhibited efficient CHK1 degradation upon dTAG^V^-1 treatment. In the absence of CHK1, there was a marked accumulation of γH2AX and phosphorylated RPA2 at both S4/S8 and S33 sites, with or without HU treatment, far exceeding the HU-induced responses observed in parental 293A cells. These findings indicate that CHK1-depleted cells accumulate extensive DNA damage and are unable to effectively cope with HU-induced replication stress.

These observations are consistent with the comet assay results shown in Fig. 2A, B, which demonstrate widespread DNA strand breaks following CHK1 depletion. Total RPA2 protein levels remained unchanged, confirming that the observed increases in phosphorylation were not due to altered protein abundance. Moreover, phosphorylation of histone H3 at Ser10 (pH3S10), a marker of mitotic entry, was absent in HU-treated 293A cells and in dTAG-CHK1 cells treated with dTAG^V^-1, either in the presence or absence of HU. This indicates that cells fail to progress into mitosis under conditions of replication stress or upon acute CHK1 depletion.

To further assess molecular markers of replication stress and DNA damage, we performed immunoblot analyses in parental 293A and dTAG-CHK1 cells treated with dTAG^V^-1 for multiple time points between 0 and 24 h. In dTAG-CHK1 cells, CHK1 protein levels became undetectable within 2 h of dTAG^V^-1 treatment, confirming efficient and rapid degradation of CHK1 under these conditions (Fig. 2D). Concomitantly, levels of γH2AX and phosphorylation of RPA2 at both S4/S8 and S33 increased in a time-dependent manner following CHK1 depletion, consistent with progressive accumulation of DNA damage.

In parallel, phosphorylation of histone H3 at Ser10 (pH3(S10)) was markedly reduced after 8 h of CHK1 degradation, although a faint signal was still detectable by immunoblotting. To more accurately determine whether CHK1-depleted cells failed to enter mitosis, we performed pH3S10 staining followed by flow cytometric analysis. As shown in Fig. S1A, the pH3S10-positive cell population began to decline at 8 h and was completely lost by 12 h following dTAG^V^-1 treatment, confirming a defect in mitotic entry upon acute CHK1 depletion.

Since CHK1-depleted cells failed to enter mitosis, we next investigated whether loss of CHK1 affects DNA replication. We first performed EdU incorporation assays to examine DNA synthesis following CHK1 degradation. In dTAG-CHK1 cells, EdU incorporation remained readily detectable at early time points (4–8 h) after dTAG^V^-1 treatment, but declined markedly by 12–16 h and was almost completely abolished by 24 h (Fig. 2E). These data indicate that although CHK1-depleted cells are able to enter S phase and initiate DNA replication, DNA synthesis becomes progressively inhibited over time, likely as a consequence of the extensive DNA damage observed in these cells. In contrast, EdU incorporation was not affected in parental 293 A control cells (Fig. 2E).

We next examined cell-cycle progression using dual EdU/FxCycle staining followed by flow cytometric analysis (Fig. 2F). In dTAG-CHK1 cells, the S-phase population progressively accumulated, particularly at 8 h and later following dTAG^V^-1 treatment. Notably, a reduction in EdU signal was already evident by 4 h post-treatment. This reduction was initially most apparent in cells in mid-to-late S phase but subsequently became evident across the entire S-phase population. Moreover, CHK1-depleted cells failed to progress into G2/M phase. Together, these results indicate that although CHK1-depleted cells can enter S phase, DNA synthesis becomes severely compromised over time, likely due to replication fork stalling and extensive DNA damage following acute CHK1 loss.

To further validate the observations described above, we treated parental 293A cells with the CHK1 inhibitor rabusertib and compared the resulting phenotypes with those induced by dTAG^V^-1-mediated CHK1 degradation in dTAG-CHK1 cells. We first determined an appropriate concentration of rabusertib using CellTiter-Glo viability assays. As shown in Fig. S1B, treatment with 20 μM rabusertib for 24 h resulted in a reduction in cell viability comparable to that observed in dTAG-CHK1 cells treated with dTAG^V^-1 for the same duration. Based on this analysis, 293A cells were treated with 20 μM rabusertib and harvested at multiple time points for immunoblotting.

As shown in Fig. S1C, pharmacological inhibition of CHK1 largely recapitulated the key phenotypes observed following genetic CHK1 degradation. Specifically, we observed a time-dependent accumulation of DNA damage markers, including increased γH2AX and phosphorylation of RPA2 at S4/S8 and S33, closely resembling the pattern observed in dTAG-CHK1 cells treated with dTAG^V^-1 (Fig. 2D). In addition, pH3(S10) levels were markedly reduced after 8 h of rabusertib treatment (Fig. S1C), indicating a defect in mitotic entry similar to that observed upon CHK1 depletion. Collectively, these results demonstrate that both genetic depletion and pharmacological inhibition of CHK1 induce replication stress, DNA damage accumulation, impaired DNA synthesis, and failure to enter mitosis.

To determine whether the effects of CHK1 degradation are generalizable across different cell types, we extended our analysis to HeLa and RPE-1 cells. HeLa cells are widely used cancer cells characterized by high proliferative capacity, whereas RPE-1 cells are non-transformed, hTERT-immortalized epithelial cells that represent a more physiologically normal cellular context. These two cell lines were selected to assess whether CHK1’s function is broadly required for genome integrity in both transformed and non-transformed backgrounds.

We successfully generated HeLa dTAG-CHK1 and RPE-1 dTAG-CHK1 cell lines, in which the CHK1 protein was efficiently degraded upon dTAG^V^-1 treatment (Fig. S2A) and resulted in cell lethality (Fig. S2B). To further evaluate the requirement for CHK1 in genome maintenance, these cells were treated with dTAG^V^-1 and harvested at multiple time points for immunoblotting and EdU incorporation analyses. As shown in Fig. S2C, D, CHK1 degradation in both HeLa and RPE-1 cells led to a time-dependent accumulation of DNA damage markers, including γH2AX and phosphorylated RPA2, closely resembling the response observed in 293A dTAG-CHK1 cells. Consistently, EdU incorporation progressively declined in both cell types following CHK1 depletion (Fig. S2E), indicating impaired DNA synthesis and replication stress similar to that observed in 293A cells (Fig. 2E). These results demonstrate that CHK1 is essential for maintaining replication fidelity and genome stability across diverse human cell types.

Together, these findings indicate that CHK1-depleted cells accumulate in S phase and fail to progress efficiently through the cell cycle, consistent with previous reports establishing an essential role for CHK1 in replication fork stability and cell-cycle progression. Importantly, our study further defines the temporal nature of these defects and reveals that acute CHK1 degradation induces widespread DNA damage even in the absence of exogenous DNA damage or replication stress.

Collectively, these data demonstrate that CHK1 is essential for maintaining DNA replication integrity and preventing replication-associated DNA damage. Acute CHK1 depletion results in replication fork collapse, accumulation of DNA lesions, and disruption of cell-cycle progression, ultimately leading to cell lethality. Notably, even in the absence of exogenous DNA-damaging agents or replication stress, the majority of dTAG-CHK1 cells lost viability following treatment with 1 μM dTAG^V^-1 for 24 h (Fig. 1F). The rapid onset of cell death suggests that CHK1 function is continuously required during S-phase progression and that its acute loss triggers rapid cell-cycle arrest and lethality.

### CHK1 depletion in S phase induces DNA damage, cell cycle arrest, and cell death

**Fig. 3:**
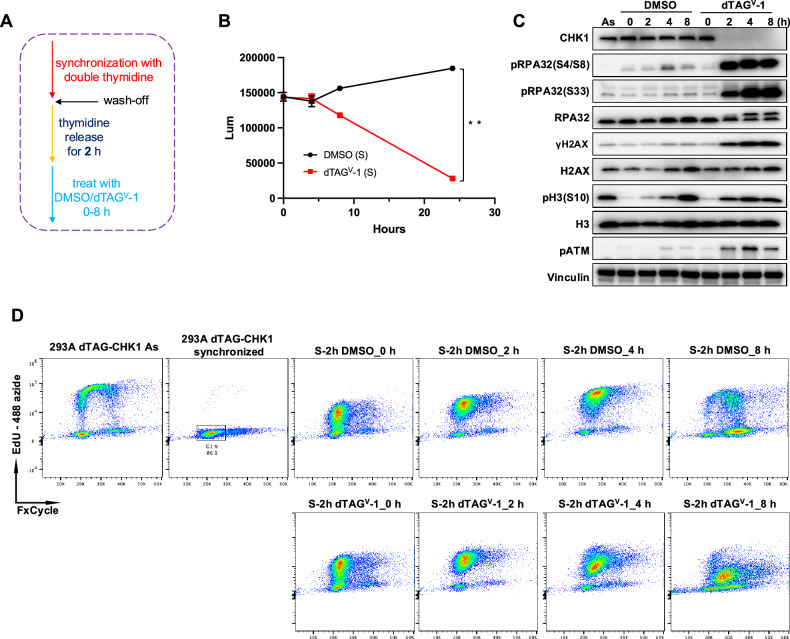
CHK1 depletion during S phase induces DNA damage responses and cell-cycle arrest. **A** Schematic illustration of the synchronization and treatment protocol. dTAG-CHK1 cells were synchronized at the G1/S boundary using a double-thymidine block and released into early S phase for 2 h before treatment with dTAG^V^-1 or DMSO. **B** Cell viability analysis of synchronized dTAG-CHK1 cells following dTAG^V^-1 or DMSO treatment for the indicated durations, measured by a luminescence-based ATP assay (*n* = 3). *P* < 0.05. **C** Immunoblot analysis showing induction of DNA damage and replication stress markers, as well as alterations in cell-cycle-associated proteins, following CHK1 depletion during S phase. **D** Cell-cycle analysis by flow cytometry (FxCycle versus EdU) showing S-phase progression and arrest in synchronized dTAG-CHK1 cells treated with DMSO or dTAG^V^-1.

To investigate the consequences of CHK1 depletion during S phase, we synchronized dTAG-CHK1 cells at the G1/S boundary using a double thymidine block. Cells were then released into S phase for 2 h before treatment with dTAG^V^-1 to induce CHK1 degradation (Fig. 3A). Cell viability analysis revealed a significant decrease in luminescence signal, reflecting reduced cell number, over time in dTAG^V^-1-treated cells compared with DMSO-treated controls, indicating cell death following CHK1 loss (Fig. 3B).

Immunoblot analysis confirmed efficient depletion of CHK1 upon dTAG^V^-1 treatment and revealed a marked increase in replication stress markers, including phosphorylation of RPA2 at S4/S8 and S33 (Fig. 3C). In addition, γH2AX levels were elevated following CHK1 degradation, consistent with the accumulation of DNA damage. Phosphorylation of ATM (pATM), a central mediator of the DNA damage response, was also increased.

To further assess the effects of CHK1 depletion on cell-cycle progression, we performed flow cytometric analysis of DNA content following dTAG^V^-1 treatment. As shown in Fig. 3D, synchronized DMSO-treated control cells progressed through mid- to late S phase, entered G2/M, and subsequently returned to G1, indicating successful completion of DNA replication and cell-cycle progression. In contrast, S-phase-synchronized cells treated with dTAG^V^-1 displayed a pronounced accumulation of cells with an S-phase-dominant DNA content profile, accompanied by a substantial reduction in EdU incorporation. These results indicate that CHK1-depleted cells experience difficulty progressing through mid- to late S phase.

Given that 293A cells are transformed and harbor inactivated p53, we next sought to validate these findings in a p53-proficient system using RPE-1 dTAG-CHK1 cells. RPE-1 dTAG-CHK1 cells were synchronized in G1 using a CDK4/6 inhibitor and then treated with DMSO or dTAG^V^-1 for an additional 2 h to induce CHK1 degradation while maintaining G1 arrest. Cells were subsequently released from G1 by washing and replacing the medium with fresh DMSO- or dTAG^V^-1-containing medium and harvested at 0-24 h post-release. Cell-cycle analysis revealed that CHK1-depleted RPE-1 cells exhibited impaired EdU incorporation during mid- to late S phase, consistent with defective DNA replication following acute CHK1 loss (Fig. S3A).

This S-phase arrest phenotype is consistent with the established role of CHK1 in stabilizing replication forks and coordinating origin firing during DNA synthesis. The accumulation of cells with S-phase DNA content (between 2 N and 4 N), accompanied by a markedly reduced EdU signal (Fig. 2D), indicates that synchronized cells that have already initiated DNA replication are unable to efficiently complete S-phase progression following CHK1 loss. This defect is likely attributable to replication fork stalling in the context of extensive DNA damage. These findings reinforce the conclusion that CHK1 is indispensable for proper S-phase progression and genome maintenance even in the absence of exogenous DNA damage or replication stress. Acute CHK1 depletion disrupts cell-cycle progression by inducing DNA breaks, stalling replication forks, and preventing successful cell-cycle transitions, ultimately leading to cell lethality.

### CHK1 loss at the G1/S boundary induces replication stress through aberrant origin activation

**Fig. 4:**
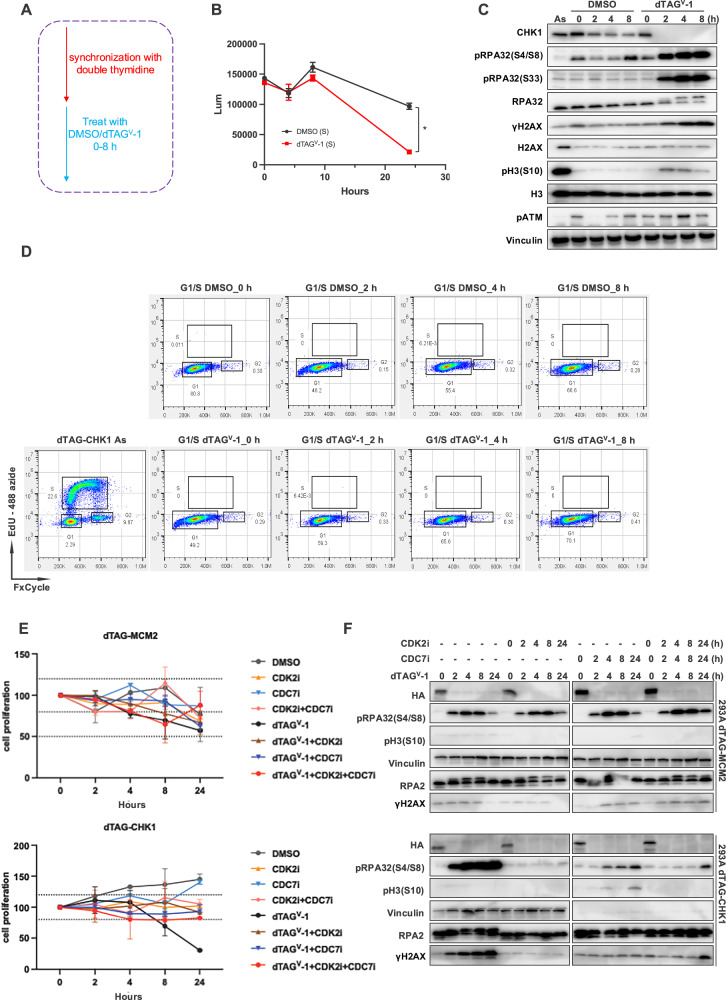
CHK1 depletion in cells arrested at the G1/S boundary induces DNA damage responses and cell death. **A** Schematic illustration of the synchronization and treatment strategy. Cells were synchronized at the G1/S boundary using a double-thymidine block and treated with DMSO or dTAG^V^-1 for the indicated durations without release into S phase. **B** Cell viability of G1/S-synchronized dTAG-CHK1 cells measured by a luminescence-based CellTiter-Glo assay following treatment with DMSO or dTAG^V^-1 (*n* = 3). *p* < 0.05. **C** Immunoblot analysis showing induction of DNA damage and replication stress markers following CHK1 depletion in G1/S-arrested cells treated with dTAG^V^-1 for 0–8 h without release. **D** Flow cytometry analysis of EdU incorporation showing cell-cycle profiles of G1/S-synchronized dTAG-CHK1 cells treated with DMSO or dTAG^V^-1 for 0–8 h, demonstrating a lack of S-phase progression. **E** Cell viability analysis of G1/S-synchronized dTAG-MCM2 and dTAG-CHK1 cells treated with dTAG^V^-1 in the presence or absence of CDK2 inhibitor (CDK2i), CDC7 inhibitor (CDC7i), or combined treatment for 0-24 h. **F** Immunoblot analysis of DNA damage markers in G1/S-synchronized dTAG-MCM2 and dTAG-CHK1 cells treated with dTAG^V^-1 with or without CDK2i and/or CDC7i for 0–24 h.

Under physiological conditions, the G1/S boundary is generally considered a pre-replicative state, in which origin licensing has occurred but bulk DNA synthesis has not yet started. We therefore initially hypothesized that CHK1 degradation at this stage would not induce substantial DNA damage or cell death, as active DNA replication, often a primary source of replication stress, would not yet be engaged. Unexpectedly, however, treatment of G1/S-synchronized cells with dTAG^V^-1 to induce acute CHK1 degradation (Fig. 4A) resulted in a clear reduction in cell viability (Fig. 4B).

To further substantiate this finding, we performed rescue experiments in G1/S-synchronized dTAG-CHK1 cells reconstituted with CHK1-FL, CHK1-FL(KD), CHK1-ND, CHK1-ND(KD), CHK1-S317A/S345A, or CHK1-SQ/AQ. Notably, only re-expression of full-length WT CHK1 effectively rescued cell viability, whereas none of the CHK1 mutants restored survival or prevented CHK1 depletion-induced cell death or arrest in double thymidine-blocked cells within 24 h (Fig. S3B). These results provide strong evidence that functional CHK1 is essential at the G1/S boundary, even prior to bulk DNA synthesis.

Consistent with the observed reduction in cell viability, immunoblot analysis revealed pronounced accumulation of DNA damage markers, including phosphorylated RPA2 and γH2AX, following CHK1 depletion (Fig. 4C). These findings indicate that even when cells are synchronized at the G1/S boundary, CHK1 plays a critical role in restraining aberrant replication initiation or inappropriate fork-associated events and in preserving genome integrity. Notably, cells remained arrested at the G1/S boundary throughout the experiment due to continued thymidine block and were not released into S phase. Flow cytometric analysis confirmed that CHK1-depleted cells did not progress into S phase or initiate detectable DNA synthesis (Fig. 4D). Nevertheless, despite the absence of bulk DNA replication, these cells continued to accumulate DNA damage following CHK1 loss (Fig. 4C).

These observations challenge the prevailing view that CHK1 functions exclusively at active replication forks during ongoing DNA synthesis. Instead, they raise the question of how CHK1 contributes to genome stability in cells arrested at the G1/S boundary, where canonical DNA replication has not yet started.

To provide further mechanistic insight into the role of CHK1 at the G1/S boundary, we synchronized control dTAG-CHK1 cells or dTAG-CHK1 cells reconstituted with either full-length WT CHK1 (CHK1-FL) or the kinase-dead mutant CHK1-FL(KD; D130A) at the G1/S boundary using a double thymidine block. Cells were treated with DMSO or dTAG^V^-1 for 0–8 h without release from the block (Fig. S4A). Immunoblot analysis revealed a progressive accumulation of DNA damage markers in cells reconstituted with the kinase-dead CHK1 mutant, whereas cells expressing WT full-length CHK1 were largely protected from DNA damage accumulation under these conditions (Fig. S4B). These results indicate that CHK1 catalytic activity is required to maintain genome stability even in cells arrested at the G1/S boundary.

We next asked whether this G1/S-specific function of CHK1 depends on ATR-mediated phosphorylation. To address this question, we performed analogous synchronization experiments using dTAG-CHK1 cells reconstituted with either WT full-length CHK1 or the CHK1-S317A/S345A mutant. Immunoblot analysis demonstrated increased DNA damage in cells expressing the phosphorylation-deficient CHK1-S317A/S345A mutant, but not in cells reconstituted with WT CHK1 (Fig. S4C). These findings suggest that the genome-protective function of CHK1 at the G1/S boundary requires ATR-dependent phosphorylation.

One possible explanation for these observations is that CHK1 may be required to suppress aberrant origin firing, particularly the unwinding activity of the replicative helicase complex (i.e., the CMG/MCM complex), even in the absence of bulk DNA replication. If this model is correct, inhibition of MCM helicase activity would be expected to attenuate the DNA damage induced by CHK1 loss. However, direct pharmacological inhibitors of the MCM helicase are not currently available. We recently generated MCM2-dTAG cells and demonstrated that acute depletion of MCM2 results in complete inhibition of DNA replication [26]. We therefore utilized this system to test whether removal of MCM2 could suppress DNA damage induced by CHK1 depletion.

MCM2-dTAG cells were synchronized at the G1/S boundary using a double thymidine block and were not released from the block, thereby remaining arrested at the G1/S boundary during MCM2 depletion. Unexpectedly, depletion of MCM2 under these conditions also resulted in increased DNA damage, as indicated by elevated phosphorylation of RPA2 (Fig. 4F). Notably, the pRPA2 signal in MCM2-depleted cells increased at early time points but subsequently declined by 24 h. In contrast, phosphorylation of RPA2 in CHK1-depleted cells continued to accumulate over time, ultimately correlating with a pronounced loss of cell viability (Fig. 4E, F). These findings suggest that the DNA damage response elicited by CHK1 depletion is mechanistically distinct from that observed following MCM2 depletion.

Based on these observations, we hypothesize that CHK1 functions to suppress inappropriate origin firing and/or restrain MCM helicase activity. Accordingly, CHK1 depletion may lead to unscheduled origin activation and/or aberrant DNA unwinding by the MCM helicase, resulting in the progressive accumulation of single-stranded DNA and DNA breaks that ultimately compromise cell viability. Although direct pharmacological inhibitors of the MCM helicase are not available, activation of the MCM complex during replication initiation requires CDK2 and CDC7 kinase activities.

To test whether the DNA damage induced by CHK1 loss is linked to origin firing and/or MCM helicase activity, we treated cells with the selective CDK2 inhibitor tagtociclib (10 μM; also known as PF-07104091), the CDC7 inhibitor (CDC7i) simurosertib (10 μM; also known as TAK-931), or a combination of both inhibitors to suppress replication initiation. We reasoned that if the DNA damage induced by CHK1 depletion depends on unscheduled origin firing and/or MCM helicase activity, inhibition of CDK2 and/or CDC7 would attenuate this phenotype. Conversely, if DNA damage occurs independently of replication initiation, these inhibitors would be expected to have little or no effect.

In these experiments, cells were treated with dTAG^V^-1 and/or the indicated kinase inhibitors during the thymidine block, without release, ensuring that all treatments occurred at the G1/S boundary (Fig. 4E). Under these conditions, dTAG-MCM2 cells did not exhibit a substantial reduction in viability within the 24-h time window, regardless of treatment with CDK2 inhibitor (CDK2i), CDC7i, or their combination.

In contrast, dTAG-CHK1 cells displayed a pronounced loss of viability upon CHK1 degradation (Fig. 4E). Notably, this survival defect was significantly rescued by co-treatment with CDK2i, CDC7i, or both inhibitors. These findings support the notion that CHK1 functions to suppress inappropriate origin firing and/or DNA unwinding by the MCM helicase at the G1/S boundary, and that loss of CHK1 leads to aberrant DNA unwinding that can be mitigated by blocking replication initiation.

To further investigate the molecular basis of these effects, we analyzed DNA damage markers under the same treatment conditions by immunoblotting (Fig. 4F). In dTAG-MCM2 cells, MCM2 depletion resulted in a modest increase in pRPA2(S4/S8) levels but did not induce a detectable increase in γH2AX within 24 h. This observation is consistent with the idea that MCM2 depletion in G1/S-arrested cells may expose limited regions of single-stranded DNA at licensed replication origins, generating a relatively mild replication-associated stress that does not lead to extensive DNA breaks or loss of cell viability (Fig. 4E). As expected, the modest pRPA2 signal induced by MCM2 depletion was not suppressed by treatment with CDK2i, CDC7i, or both.

By contrast, CHK1 degradation induced robust and progressive accumulation of both pRPA2(S4/S8) and γH2AX in cells arrested at the G1/S boundary (Fig. 4F), indicating sustained generation of single-stranded DNA and DNA breaks over time. Importantly, treatment with CDK2i, CDC7i, or their combination markedly suppressed the accumulation of these DNA damage markers. Together, these results support the conclusion that CHK1 plays a critical and non-redundant role in restraining origin firing and maintaining genome stability during the G1/S transition.

Collectively, these data indicate that CHK1 is required not only for resolving replication stress during S phase but also for preventing aberrant origin firing and/or DNA unwinding at the G1/S boundary. Acute loss of CHK1, even in the absence of bulk DNA synthesis, is therefore sufficient to trigger DNA damage and ultimately compromise cell viability.

To further establish the importance of ATR and CHK1 activities in normal S-phase progression, we treated 293A cells with prexasertib, another ATP-competitive small-molecule inhibitor of CHK1. Treatment with relatively low doses of prexasertib significantly reduced the growth of 293A cells (Fig. S5A). Consistently, prexasertib-treated cells exhibited defective EdU incorporation during S-phase progression, indicating impaired DNA synthesis (Fig. S5B). Similarly, treatment with the ATR inhibitor gartisertib led to a marked reduction in cell viability (Fig. S5C) and a clear induction of DNA damage, as evidenced by increased DNA damage markers (Fig. S5D). Flow cytometric analysis further revealed defective EdU incorporation in gartisertib-treated cells during S phase (Fig. S5E). These observations are consistent with prior work from Zha and colleagues demonstrating an essential role for ATR in S-phase progression [27].

**Fig. 5:**
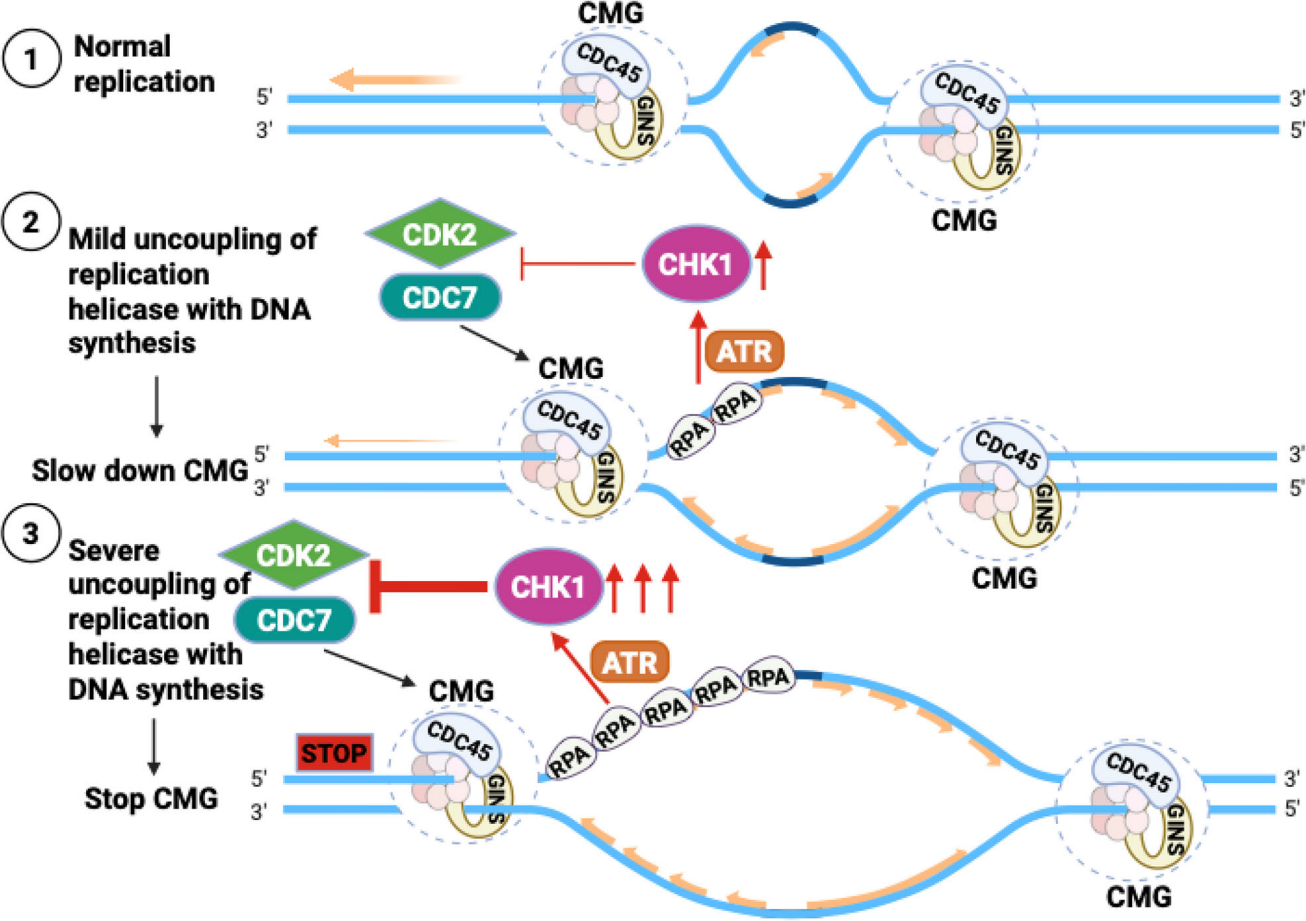
CHK1 is an integral regulator of DNA replication in human cells. (1) During normal DNA replication, CHK1 ensures proper coupling between the CMG helicase and DNA polymerases through a feedback regulatory mechanism that maintains coordinated fork progression. (2) Moderate ATR-mediated activation of CHK1 is induced by partial uncoupling of the replicative helicase from DNA synthesis, which results in slowed CMG helicase progression to stabilize replication forks. (3) Strong ATR-mediated activation of CHK1 resulted from pronounced uncoupling of the replicative helicase from DNA synthesis, effectively halting CMG helicase activity to prevent replication-associated genome instability.

Together, our results demonstrate that CHK1 is indispensable not only for S-phase progression during normal DNA replication in the absence of exogenous stress, but also for maintaining genome stability at the G1/S transition (Fig. 5). These findings highlight a previously underappreciated role for CHK1 in safeguarding genome integrity from the very onset of DNA replication.

## Discussion

Our results confirm that acute CHK1 depletion leads to rapid cell death, consistent with prior studies demonstrating that CHK1 inhibition disrupts replication stress management and checkpoint signaling [4, 5, 28]. However, in contrast to pharmacological inhibitors that may permit residual CHK1 activity, the dTAG system enables rapid and near-complete elimination of CHK1, thereby revealing a more pronounced and immediate impact on DNA replication and cell-cycle progression.

We observed that CHK1-depleted cells accumulated extensive DNA damage within h, as evidenced by γH2AX induction and RPA2 phosphorylation, paralleling phenotypes reported in genetic CHK1 knockout models [8]. Importantly, our rescue experiments demonstrated that only full-length, catalytically active CHK1 was capable of restoring cell viability, reinforcing the conclusion that both CHK1 kinase activity and ATR-mediated phosphorylation are indispensable for cell survival.

Consistent with previous studies, CHK1 depletion resulted in replication fork collapse and cell-cycle arrest, phenotypes that have been widely observed in CHK1-inhibited cells under conditions of replication stress [29]. Notably, our findings extend this paradigm by demonstrating that CHK1 loss alone, even in the absence of exogenous DNA damage or replication stress, is sufficient to trigger replication catastrophe and cell death. This observation suggests that the role of CHK1 in maintaining replication integrity is more fundamental than previously appreciated.

A key insight from our study is that CHK1 exerts an essential preemptive function at the G1/S boundary. We show that acute CHK1 loss at this stage leads to accumulation of single-stranded DNA and DNA damage despite the absence of bulk DNA synthesis, indicating that CHK1 restrains aberrant origin firing and/or inappropriate DNA unwinding before the onset of S phase. These findings underscore a critical role for CHK1 in safeguarding genome stability from the very beginning of DNA replication.

In conclusion, our study provides a detailed mechanistic understanding of CHK1 function during normal DNA replication and cell-cycle progression. By leveraging the temporal precision of the dTAG system, we demonstrate that CHK1 depletion results in replication fork collapse, DNA damage accumulation, and rapid cell death, thereby reinforcing the central role of CHK1 in maintaining cell viability and genome integrity.

## Materials and methods

### Cell culture and transfection

HEK293A (293A) and HEK293T (293T) cells were obtained from the American Type Culture Collection (ATCC) and maintained in Dulbecco’s modified Eagle medium (DMEM) supplemented with 10% fetal calf serum (FCS) at 37 °C in a humidified incubator with 5% CO₂. hTERT RPE-1 (RPE-1) cells were a kind gift from Dr. Hendrickson’s laboratory at the University of Virginia and were cultured in DMEM/F-12 supplemented with 10% FCS and 0.01 mg/mL hygromycin B. All cell lines were routinely tested and confirmed to be negative for mycoplasma contamination.

Transient plasmid transfections were performed using polyethylenimine (PEI). Briefly, cells were seeded onto 6-well plates 16–18 h prior to transfection. For each well, 6 μg of PEI and 2 μg of plasmid DNA were diluted separately in 100 μL of Opti-MEM Reduced Serum Medium (Life Technologies). The diluted PEI and DNA solutions were then combined and incubated at room temperature for 15 min to allow complex formation. The resulting transfection mixture was subsequently added dropwise to the cells.

### Virus production and transduction

Lentiviral particles were produced in HEK293T cells by co-transfecting pMD2.G (Addgene #12259), psPAX2 (Addgene #12260), and the indicated lentiviral expression plasmids using X-tremeGENE HP DNA transfection reagent (Sigma-Aldrich, #06366546001), according to the manufacturer’s instructions. Viral supernatants were collected 48 h after transfection and filtered through a 0.45 μm membrane. Filtered viral supernatants were applied to 293A cells in the presence of 10 μg/mL polybrene. Transduced cells were selected using 2 μg/mL puromycin (puromycin dihydrochloride; Thermo Fisher Scientific, A1113803).

Recombinant adeno-associated virus (rAAV) was produced in AAV-293 cells by co-transfecting pAAV-RC and pAAV-helper (both gifts from Dr. Hendrickson’s laboratory) together with the pAAV-MCS-GG-YFG donor plasmid using X-tremeGENE HP DNA transfection reagent (Sigma-Aldrich, #06366546001), following the manufacturer’s protocol. Forty-eight h after transfection, cells were scraped, collected in 1 mL of culture medium, and transferred to a 1.5 mL microcentrifuge tube. Cells were subjected to three freeze-thaw cycles using a dry ice/ethanol bath, with vortexing between each cycle. Cell debris was pelleted by microcentrifugation, and the clarified supernatant was filtered through a 0.45 μm membrane. For initial transduction experiments, increasing volumes of rAAV (ranging from 1–2 μL up to 200 μL) were tested.

### Generation of CHK1 inducible KO cells (i.e., dTAG-CHK1 293A/HeLa cells)

dTAG-CHK1 cells were generated by knocking in a Blasticidin-P2A-2×HA-FKBP12^F36V^ –linker cassette (dTAG) at the N terminus of the endogenous CHK1 gene. A CHK1 N-terminal-specific guide RNA (gRNA) expression vector was constructed by inserting the gRNA sequence (CHK1-N-gRNA2: AGTGCCCTTTGTGGAAGACT), located near the CHK1 ATG start codon, into the PX330 vector (Addgene #42230). The genome-editing efficiency of the CHK1-N-gRNA2 construct was evaluated using a T7 endonuclease I assay (New England Biolabs, M0302S) according to the manufacturer’s instructions.

To isolate genomic DNA for the generation of homologous arms, cells were washed with cold PBS and lysed using QuickExtract DNA Extraction Solution 1.0 (Epicenter, QE09050). Cell lysates were incubated at room temperature for 10 min with shaking, transferred to PCR tubes, and subsequently incubated at 65 °C for 15 min followed by 95 °C for 5 min. The 5′ and 3′ homology arms of CHK1 were amplified by PCR using genomic DNA as the template, and the Blasticidin-P2A-2×HA-FKBP12^F36V^-linker cassette was amplified by PCR from pCRIS-PITChv2-BSD-dTAG (BRD4) (Addgene #91792). These fragments were assembled into the pUC19 backbone using Gibson Assembly Master Mix (New England Biolabs) following the manufacturer’s protocol to generate the dTAG-CHK1 knock-in donor plasmid (pUC19-dTAG-CHK1-NKI-donor). All primers were designed with overlapping sequences to facilitate Gibson assembly and were synthesized by Integrated DNA Technologies.

PX330-CHK1-N-gRNA2 and pUC19-dTAG-CHK1-NKI-donor plasmids were co-transfected into 293A or HeLa cells by transient transfection. Transfected cells were selected using 10 μg/mL blasticidin (blasticidin S HCl; Thermo Fisher Scientific, A1113903) and subsequently seeded at a density of one cell per well in 96-well plates to obtain single-cell-derived clones. Individual clones were expanded, and genomic DNA was isolated for PCR-based screening.

To verify the correct dTAG knock-in, PCR was performed using genomic DNA, PrimeSTAR Max DNA Polymerase (Takara Bio USA, R045B), and the following primers: CHK1-NKI-forward (GGCATGGTGGGAGAAAGTTAGCATTGACTA) and CHK1-NKI-reverse (CAAAGACGTCATTATGCTGTCACACAAGTC). PCR products were analyzed by agarose gel electrophoresis. Clones displaying only the upper PCR band, indicative of homozygous insertion of the Blasticidin-P2A-2×HA-FKBP12^F36V^-linker cassette at the CHK1 N terminus, were selected as dTAG-CHK1 293A or HeLa cell lines. Efficient degradation of CHK1 protein in these clones was further confirmed by immunoblotting following treatment with 1 μM dTAG^V^-1.

### Generation of CHK1 inducible KO cells (i.e., dTAG-CHK1 RPE1 cells)

The 5′ arm-Blasticidin-P2A-2×HA-FKBP12^F36V^-linker-3′ arm cassette was amplified by PCR using pUC19-dTAG-CHK1-NKI-donor as the template and the following primers: CHK1-LarmForward (LF), GACGCTCTTCACCGTGGTACCAGGAGGTTCCCGTTGTGGGGG; and CHK1-RarmReverse (RR), GACGCTCTTCCATGGATTGAGATAATTCTAAGCCACATCACATATATAC. The resulting PCR product was cloned into the pAAV_MCS_GG_SEPT_Neo_N2 vector using BspQI restriction endonuclease digestion followed by T4 DNA ligase-mediated ligation, according to the manufacturer’s instructions, to generate the pAAV-dTAG-CHK1 knock-in donor plasmid (pAAV-dTAG-CHK1-NKI-donor). All primers were synthesized by Integrated DNA Technologies.

For in vitro synthesis of sgRNAs, a DNA template containing a T7 promoter, a 20-nt guide sequence (CHK1-N-gRNA2: AGTGCCCTTTGTGGAAGACT), and the sgRNA scaffold was generated by overlapping PCR. sgRNAs were synthesized using the EnGen® sgRNA Synthesis Kit, S. pyogenes (New England Biolabs, E3322V/S). Briefly, EnGen 2× sgRNA Reaction Mix, target-specific DNA oligonucleotide (1 μM), DTT (0.1 M), and EnGen sgRNA Enzyme Mix were thoroughly mixed and incubated at 37 °C for 30 min. DNase I was then added, followed by incubation at 37 °C for an additional 15 min. sgRNAs were subsequently purified according to the manufacturer’s protocol or analyzed by agarose gel electrophoresis.

Human RPE-1 cells were electroporated with ribonucleoprotein (RNP) complexes containing recombinant Cas9 protein and the CHK1 gRNA. For homology-directed repair using the rAAV donor, pAAV-dTAG-CHK1-NKI-donor virus was added 30 min after electroporation, using increasing volumes of virus (2 μL, 20 μL, and 200 μL) for initial transduction optimization. Transduced cells were selected with 10 μg/mL blasticidin (blasticidin S HCl; Thermo Fisher Scientific, A1113903) and seeded at a density of one cell per well in 96-well plates to obtain single-cell-derived clones.

Individual clones were expanded, and genomic DNA was isolated for PCR-based verification using PrimeSTAR Max DNA Polymerase (Takara Bio USA, R045B) and the following primers: CHK1-NKI-forward (GGCATGGTGGGAGAAAGTTAGCATTGACTA) and CHK1-NKI-reverse (CAAAGACGTCATTATGCTGTCACACAAGTC). PCR products were analyzed by agarose gel electrophoresis, and clones displaying only the upper PCR band were selected as candidate dTAG-CHK1 RPE-1 cell lines. Efficient degradation of CHK1 protein in these clones was further confirmed by immunoblotting following treatment with 1 μM dTAG^V^-1.

### Generation of CHK1 expression constructs

The constructs pDonor-CHK1-KD, pDonor-CHK1-ND, pDonor-CHK1-NDKD, pDonor-CHK1-S317A/S345A (pDonor-CHK1-S2m), and pDonor-CHK1-S317A/S345A/S357A/S366A/S468A (pDonor-CHK1-S5m) were generated from pDonor-CHK1-FL using overlapping PCR and site-directed mutagenesis via circular plasmid amplification.

Gateway LR recombination reactions were performed using Gateway™ LR Clonase™ II Enzyme Mix (Thermo Fisher Scientific) according to the manufacturer’s instructions. Briefly, a total reaction volume of 5 μL was prepared containing the entry clone (pENTR vectors carrying pDonor-CHK1-FL, pDonor-CHK1-KD, pDonor-CHK1-ND, pDonor-CHK1-NDKD, pDonor-CHK1-S2m, or pDonor-CHK1-S5m), the destination vector (pLEX307-N-SFB), 5× LR Clonase™ II Reaction Buffer, and LR Clonase™ II Enzyme Mix. The recombination reaction was incubated at 25 °C for 1 h and subsequently transformed into *E. coli* DH5α competent cells. Bacterial colonies were screened by PCR and/or restriction enzyme digestion to confirm successful recombination.

All plasmid constructs were fully sequenced to verify insert integrity and to ensure that no unintended mutations were introduced during cloning. To validate protein expression and the presence of the SFB tag, expression constructs were transiently transfected into HEK293T cells using Lipofectamine™ 2000 (Thermo Fisher Scientific) according to the manufacturer’s protocol. Cells were harvested 48 h after transfection, and whole-cell lysates were prepared for immunoblot analysis.

### Neutral and alkaline comet assays

The single-cell gel electrophoresis comet assay (R&D Systems, 4250-050-K) was performed according to the manufacturer’s protocol with minor modifications. Briefly, 5 μL of cell suspension was mixed with 50 μL of low-melting-point agarose (LMAgarose) and immediately spread onto comet assay slides. Slides were placed at 4 °C in the dark for 30 min to allow agarose solidification and then immersed in pre-chilled lysis solution at 4 °C for 1 h in the dark. Slides were subsequently equilibrated in an electrophoresis tank containing freshly prepared alkaline solution (for alkaline comet assays: 300 mM NaOH, 1 mM EDTA, pH > 13) or Tris-borate-EDTA (TBE) buffer (for neutral comet assays: 89 mM Tris, 89 mM boric acid, and 2 mM EDTA) for 15 min.

Electrophoresis was performed at 25 V for 25 min for neutral comet assays or at 25 V for 40 min for alkaline comet assays. Following electrophoresis, slides were removed from the tank, washed in distilled water for 5 min, and fixed in 70% ethanol for 5 min. DNA was then stained with 100 μL of diluted SYBR Gold (Thermo Fisher Scientific, S11494) for 30 min in the dark. All steps were carried out under low-light conditions to minimize additional DNA damage.

Images were acquired using a Nikon Eclipse 90i microscope. DNA damage was quantified by measuring comet tail length using the OpenComet plugin for ImageJ. At least 100 cells were analyzed per slide.

### Cell proliferation and colony formation assays

For cell proliferation assays, cells were seeded into 6-well plates at a density of 40,000 cells per well and cultured in the presence of 1 μM dTAG^V^-1 or an equivalent volume of DMSO. Cell numbers were determined at the indicated time points (2, 4, 6, 8, 10, and 12 days).

For colony formation assays, cells were seeded into 6-well plates at a density of 500 cells per well, treated with 1 μM dTAG^V^-1 or DMSO, and cultured for 8–10 days. Colonies were then fixed and stained with crystal violet solution (Sigma-Aldrich, HT90132-1L) for visualization.

For experiments involving dTAG^V^-1 or DMSO treatment of defined durations, culture medium containing 1 μM dTAG^V^-1 or DMSO was added only once at the start of the experiment and maintained until the specified time point. The culture medium was not replaced, and no additional dTAG^V^-1 or DMSO was added during the course of the assay.

### Cell viability assays

For cell viability assays, 1000 cells per well were seeded into 96-well plates in 100 μL of complete culture medium and incubated for 24 h. Cells were then treated by adding 100 μL of medium containing 2 μM dTAG^V^-1 or an equivalent volume of DMSO at the indicated concentrations, resulting in a final volume of 200 μL per well, and incubated for 0–48 h. For untreated control wells, 100 μL of fresh medium was added.

At the indicated time points, culture medium was removed, and 80 μL of CellTiter-Glo reagent (Promega) was added to each well. Plates were covered and shaken at room temperature for 15 min to allow complete cell lysis and signal stabilization. Luminescence was measured using a BioTek Synergy 2 SL microplate reader. Cell viability was calculated as the percentage of relative luminescence units (RLU) in treated samples relative to untreated controls.

The CHK1 inhibitors rabusertib (LY2603618; MedChemExpress, HY-14720) and prexasertib (LY2606368; MedChemExpress, HY-18174), as well as the ATR inhibitor gartisertib (VX-803; MedChemExpress, HY-136270), were obtained from MedChemExpress.

### Western blotting analysis

Protein samples were resolved by SDS-PAGE and transferred onto polyvinylidene difluoride (PVDF) membranes (Millipore). Membranes were blocked in blocking buffer and incubated with the indicated primary antibodies, followed by washing and incubation with appropriate horseradish peroxidase-conjugated secondary antibodies. Protein signals were detected using enhanced chemiluminescence (ECL) reagents (GE Healthcare) and visualized using standard imaging systems. Vinculin was used as a loading control for total cell lysates.

### Antibodies

The following antibodies were used for Western blotting and/or immunostaining: hemagglutinin (HA) tag (Cell Signaling Technology [CST], #2999S); phospho-RPA2 (Ser4/Ser8) (Bethyl Laboratories, A300-245A); phospho-RPA2 (Ser33) (Bethyl Laboratories, A300-246A); RPA2 (CST, #2208S); phospho-CHK1 (Ser296) (CST, #90178S); phospho-CHK1 (Ser317) (CST, #12302S); phospho-CHK1 (Ser345) (CST, #2348S); CHK1 (CST, #2360S); phospho-ATM (Ser1981) (CST, #13050S); phospho-histone H2AX (Ser139; γH2AX) (CST, #9718S); histone H2AX (Bethyl Laboratories, A300-082A); phospho-histone H3 (Ser10) (Abcam, ab47297); histone H3 (Abcam, ab8898); cyclin A (Santa Cruz Biotechnology, sc-239); cyclin B (Santa Cruz Biotechnology, sc-245); FLAG tag (Sigma-Aldrich, F3165); and vinculin (Sigma-Aldrich, V9264).

### Fluorescence-activated cell sorting (FACS) and cell cycle analysis

For cell cycle analysis, asynchronous cell populations were labeled with 10 μM 5-ethynyl-2′-deoxyuridine (EdU; Sigma-Aldrich, T511285-5MG) for 30 min at 37 °C. Cells were harvested, washed with PBS, fixed, and permeabilized according to standard procedures. Incorporated EdU was detected using the Click-iT™ Cell Reaction Buffer Kit (Thermo Fisher Scientific, C10269) with Alexa Fluor™ 488 azide (Thermo Fisher Scientific, A10266), following the manufacturer’s instructions.

EdU-labeled cells were washed with 3% bovine serum albumin (BSA) in PBS and resuspended in PBS containing FxCycle™ Violet stain (Thermo Fisher Scientific, F10347) and RNase A (0.1 mg/mL; Thermo Fisher Scientific, EN0531). Data were acquired by FACS using a Gallios™ 561 flow cytometer. Data analysis was performed using FlowJo v10 software.

### Statistical analysis

All experiments, including colony formation assays, cell proliferation assays, and immunofluorescence staining experiments, were performed at least three times independently. Quantitative data are presented as mean ± standard deviation (SD). Statistical significance between two groups was determined using two-tailed Student’s *t*-tests. *p* values < 0.05 were considered statistically significant and are indicated by an asterisk (*).

## Supplementary information

Supplemental Figure Legends and Supplemental Figures

Source data

## Data Availability

The data supporting the findings of this study are available from the corresponding author upon reasonable request.
