# Supplementary material for: CHK1 is an integral regulator of DNA replication in human cells
Source: Cell Death Dis. 2026 Mar 26;17(1):375. doi: 10.1038/s41419-026-08624-1 (PMC13039271; doi:10.1038/s41419-026-08624-1)
Supplement: Supplementary file 1 — Supplemental Figure Legends and Supplemental Figures [file 41419_2026_8624_MOESM1_ESM.pdf]

Supplementary Materials

Supplemental Figure S1

(A) Flow cytometry analysis showing phospho-histone H3 (Ser10; pH3(S10)) profiles in 293A-derived dTAG-CHK1 cells treated with DMSO or dTAG^{V}-1.

(B) Cell viability analysis (CellTiter-Glo assay) of parental 293A cells treated with increasing concentrations of the CHK1 inhibitor rabusertib for 24 hours to determine the working concentration.

(C) Immunoblot analysis showing induction of DNA damage markers and mitotic entry defects in 293A cells treated with 20 μM CHK1 inhibitor rabusertib for the indicated time points.

Supplemental Figure S2

(A) Generation of HeLa and RPE1 dTAG-CHK1 cell lines and confirmation of efficient CHK1 degradation following dTAG^{V}-1 treatment.

(B) Clonogenic survival assays showing loss of viability in HeLa and RPE1 dTAG-CHK1 cells upon CHK1 degradation, indicating that CHK1 is essential for cell survival in both transformed and non-transformed cell types.

(C) Immunoblot analysis of DNA damage markers and pH3(S10) in HeLa dTAG-CHK1 cells treated with dTAG^{V}-1 for the indicated time points.

(D) Immunoblot analysis of DNA damage markers and pH3(S10) in RPE1 dTAG-CHK1 cells treated with dTAG^{V}-1 for the indicated time points.

(E) Flow cytometry analysis of EdU incorporation showing replication stress and impaired S-phase progression in HeLa dTAG-CHK1 and RPE1 dTAG-CHK1 cells treated with dTAG^{V}-1 for the indicated time points.

Supplemental Figure S3

(A) Upper panel: workflow for synchronizing RPE1 dTAG-CHK1 cells in G1 phase. RPE1 dTAG-CHK1 cells were seeded onto 150-mm dishes and treated with a CDK4/6 inhibitor for 22 hours to arrest cells in G1. DMSO or dTAG^{V}-1 was then added for an additional 2 hours to degrade CHK1 while cells remained arrested in G1. Cells were subsequently released from G1 arrest by washing and replacing the medium with fresh DMSO- or dTAG^{V}-1-containing medium for 0–24 hours. Samples with pulse EdU labeling were collected at the indicated time points.
Lower panel: flow cytometry analysis of synchronized RPE1 dTAG-CHK1 cells released into DMSO- or dTAG^{V}-1-containing medium.
(B) Cell viability measured by CellTiter-Glo assay in 293A-derived dTAG-CHK1 cells and dTAG-CHK1 cells reconstituted with wild-type or mutant CHK1 constructs (FL, FLKD, ND, NDKD, S317A/S345A, or SQ/AQ). Cells were arrested at the G1/S boundary using a double-thymidine block and treated with dTAG^{V}-1 for 24 hours without release.

Supplemental Figure S4

(A) Workflow for synchronizing 293A-derived dTAG-CHK1 cells and dTAG-CHK1 cells reconstituted with CHK1-FL, CHK1-FLKD (D130A), or CHK1-S317A/S345A mutants at the G1/S boundary using a double-thymidine block. Cells were treated with DMSO or dTAG^{V}-1 for 0-8 hours without release.
(B, C) Immunoblot analysis of DNA damage and cell-cycle markers in G1/S-arrested cells following dTAG^{V}-1 treatment for the indicated time points in 293A-derived dTAG-CHK1 cells and cells reconstituted with CHK1-FL or CHK1-FLKD (D130A) (B), or with CHK1-FL or CHK1-S317A/S345A (C).

Supplemental Figure S5

(A) CellTiter-Glo assay of parental 293A cells treated with increasing concentrations of the CHK1 inhibitor prexasertib for 24 hours to determine the working concentration.

(B) Flow cytometry analysis of EdU incorporation showing replication stress and S-phase progression defects in 293A cells treated with 0.5 μM prexasertib.

(C) Cell viability analysis of parental 293A cells treated with increasing concentrations of the ATR inhibitor gartisertib for 24 hours to determine the working concentration.

(D) Immunoblot analysis showing induction of DNA damage markers and mitotic entry defects in 293A cells treated with 1 μM gartisertib for the indicated time points.

(E) Flow cytometry analysis of EdU incorporation showing replication stress and S-phase progression defects in 293A cells treated with 0.625 μM gartisertib.
