# Supplementary figures and images for: CHK1 is an integral regulator of DNA replication in human cells
Source: Cell Death Dis. 2026 Mar 26;17(1):375. doi: 10.1038/s41419-026-08624-1 (PMC13039271; doi:10.1038/s41419-026-08624-1)

Figure S1.

A

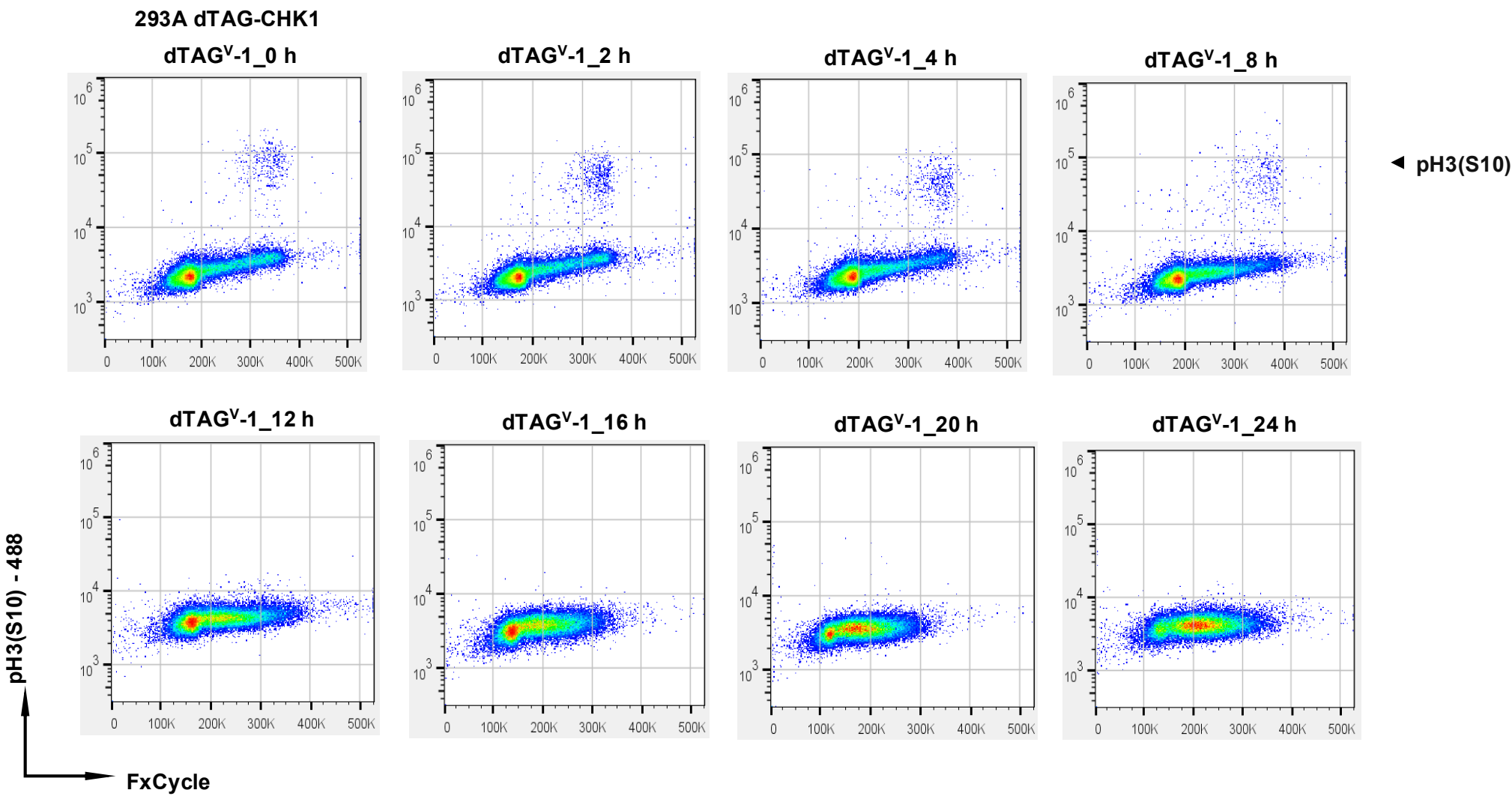

B

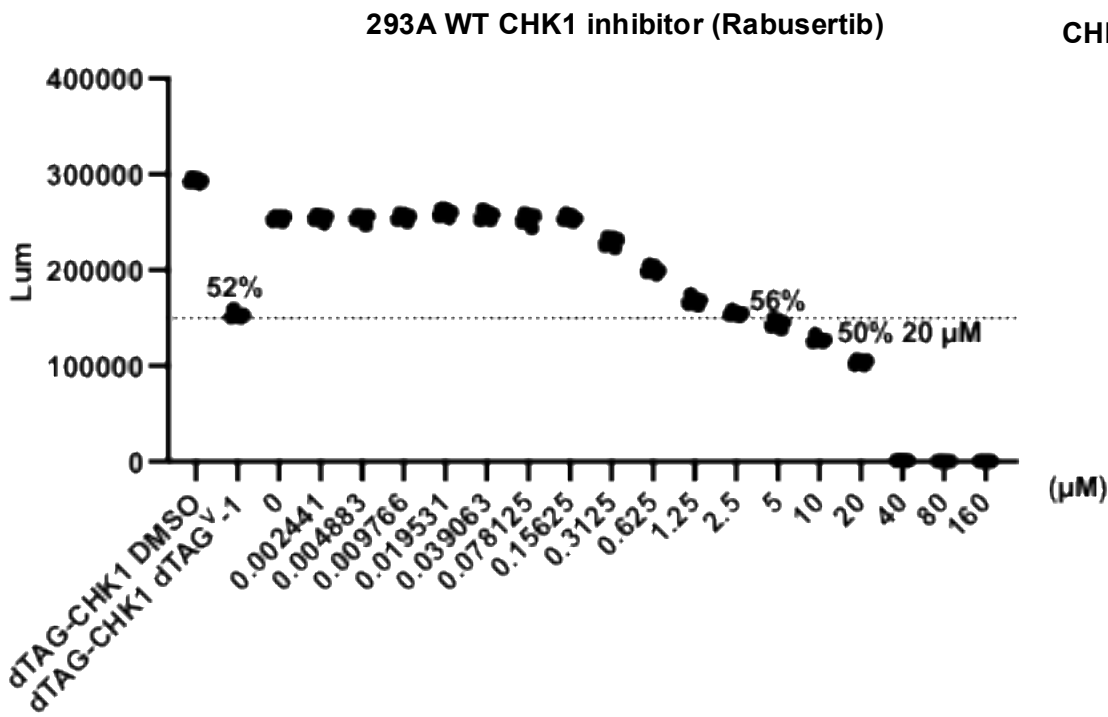

C

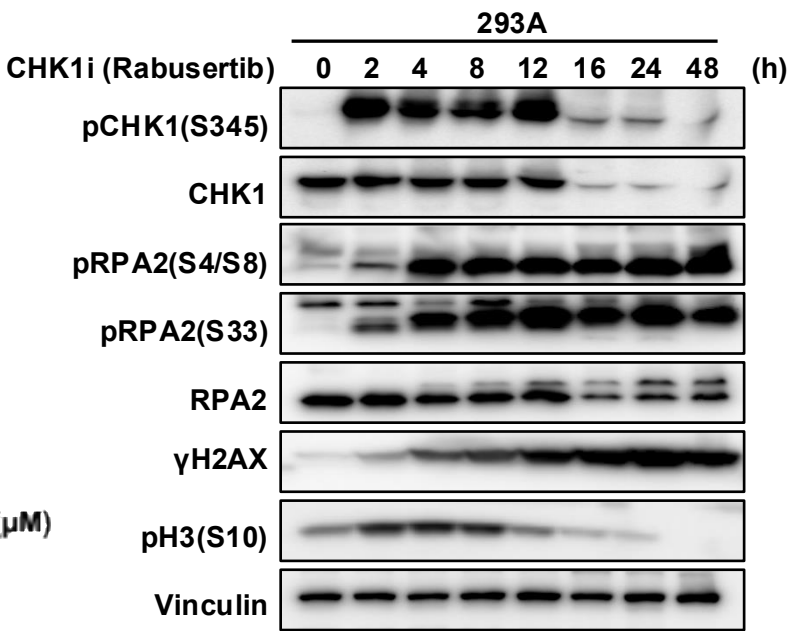

Figure S2.

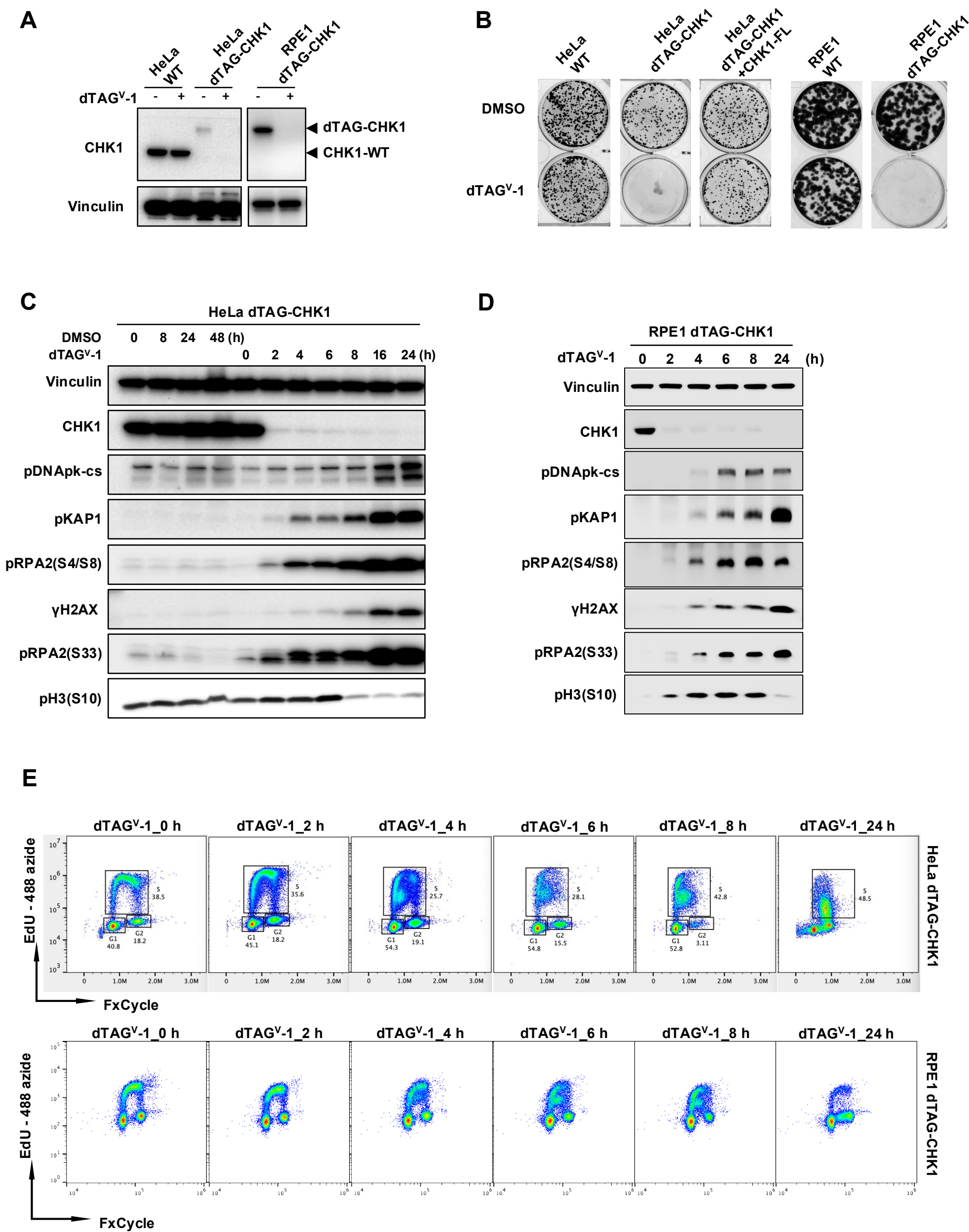

Figure S3.

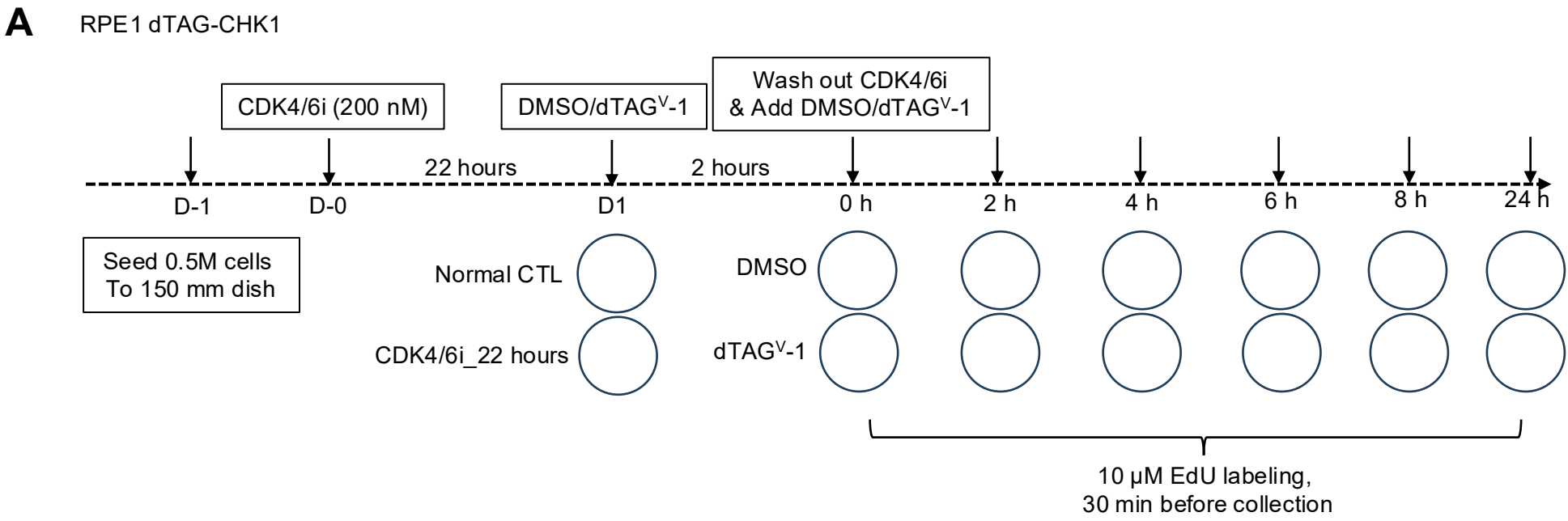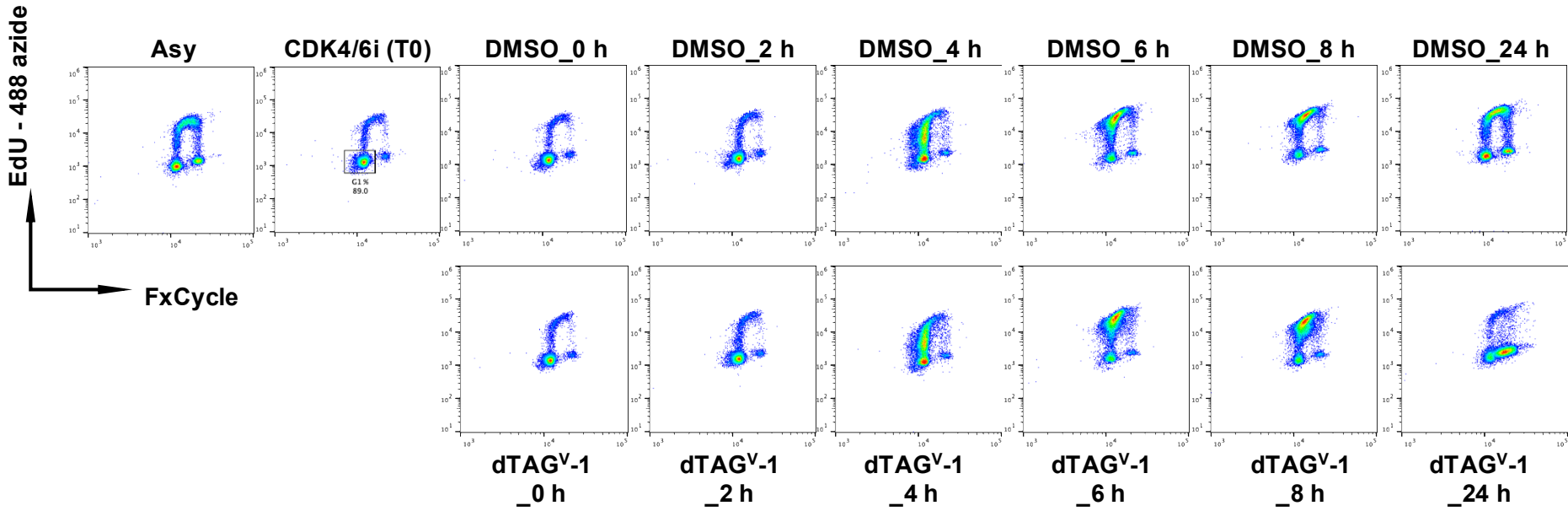

**A** 293A dTAG-CHK1

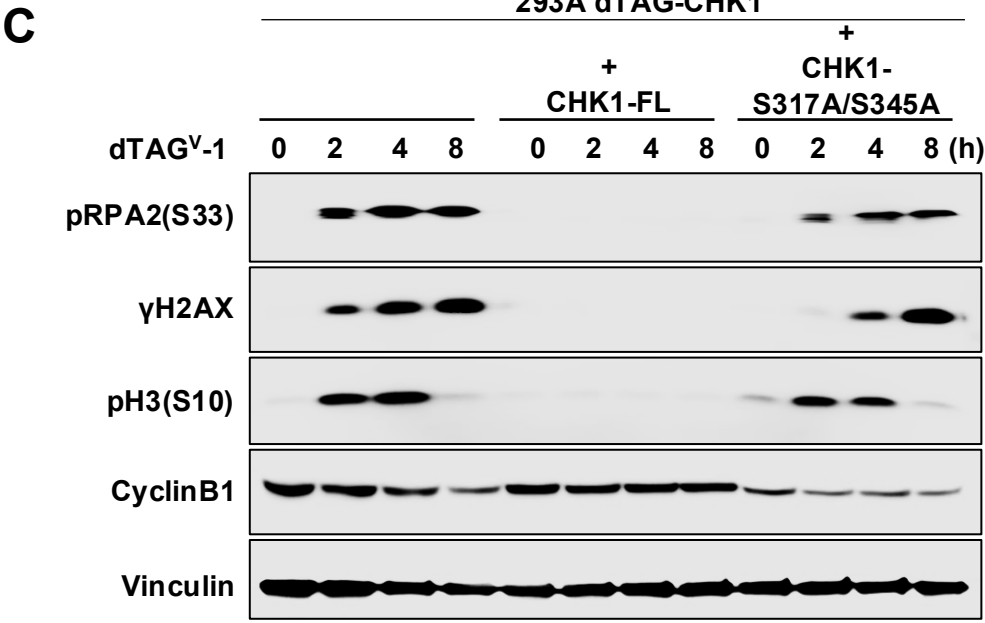

Figure S5.

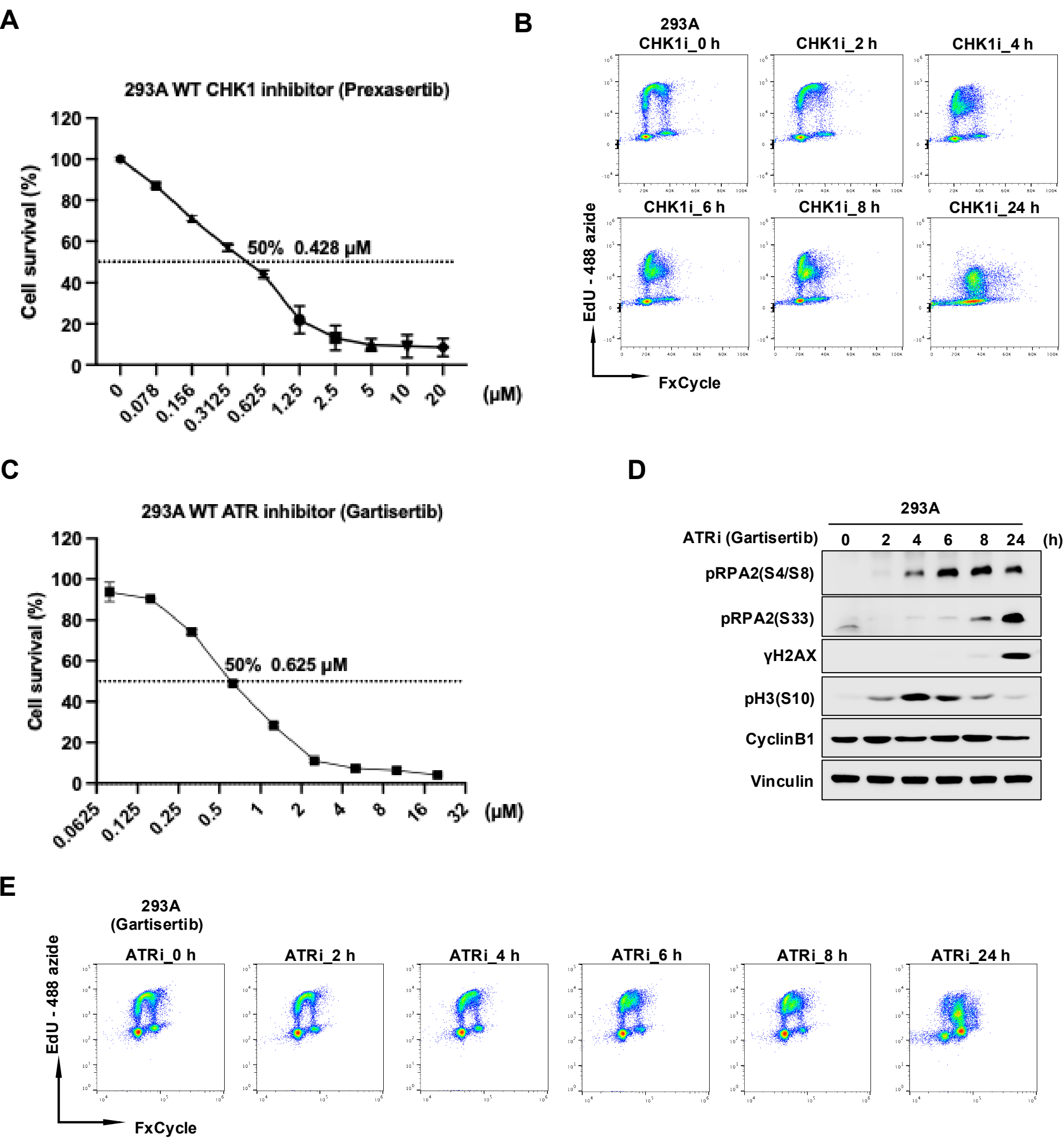

Supplement: Supplementary file 2 — Source data [file 41419_2026_8624_MOESM2_ESM.pdf]
